# Knowledge and Attitudes of Parents Regarding Neonatal Jaundice in Bisha City, Saudi Arabia

**DOI:** 10.7759/cureus.44916

**Published:** 2023-09-08

**Authors:** Jaber A Alfaifi, Youssef A Alqahtani, Masoud M Alqahtani, Anas Alqarni, Abdulmohsen Alshahrani, Raydaa A Alshomrani

**Affiliations:** 1 Pediatrics, University of Bisha, Bisha, SAU; 2 Department of Child Health, King Khalid University, Abha, SAU; 3 College of Medicine, University of Bisha, Bisha, SAU

**Keywords:** neonates, parents, bisha, knowledge, jaundice

## Abstract

Background

Neonatal jaundice (NNJ) is one of the most common neonatal illnesses around the world. It continues to be a leading cause of avoidable brain damage, physical and mental impairment, and death in neonates. Neonatal morbidity due to NNJ has a significant impact and cost on families and healthcare resources. The majority of instances are addressed without intervention, but others require assessment and assistance in the form of follow-up or treatment. Inadequate family awareness and understanding of this frequent newborn condition can contribute to delays and difficulties.

Aim

This study aimed to assess the knowledge and attitudes of parents regarding NNJ in Bisha City, Saudi Arabia.

Methodology

A cross-sectional study involving 242 participants was carried out. Data were collected using an online questionnaire. The knowledge score differences between groups were analyzed using a Wilcoxon rank sum test and the Kruskal-Wallis rank test.

Results

In this study of 242 participants from Bisha, we found that the majority were female (155, 64.0%), employed (129, 53.3%), and held a postgraduate degree (150, 62.0%). Regarding knowledge of NNJ, 109 (45.0%) have correctly defined it as yellow pigmentation in the sclera and 64 (26.4%) as yellow pigmentation of the skin. Of most participants, 132 (54.5%) recognized that NNJ could cause complications, but 53 (40.2%) of these were unaware of what these complications might be. Notably, only 89 (36.8%) of respondents believed they had sufficient knowledge about NNJ. The median knowledge score was 3.0 (IQR, 1.0-4.0), and higher scores were significantly associated with being female and reporting sufficient knowledge about the disease. Strategies suggested for improving awareness included campaigns (98, 40.5%) and social networking programs (81, 33.5%). The data suggest a need for enhanced public education regarding NNJ.

Conclusion

The study highlights the need for increased awareness and education among parents in Bisha, Saudi Arabia, about NNJ. The findings suggest that campaigns and social networking programs could effectively educate people about the condition. Additionally, the study provides insights into the factors associated with higher knowledge scores, such as gender and having a child with NNJ. The results of this study may help healthcare professionals develop effective educational programs and interventions to improve parents’ knowledge and attitudes toward NNJ.

## Introduction

Neonatal jaundice (NNJ) is one of the most common clinical conditions among newborn infants [1]. It occurs because of bilirubin accumulation in tissues, including the skin and mucus membranes, and presents as yellowish discoloration of the skin and sclera in infants. Babies with light skin tones are considered to exhibit jaundice at bilirubin levels of approximately 90 mmol/L [1]. In some countries, 75% of hospital readmissions in the first week after birth are due to NNJ [2]. NNJ occurs in approximately half to 60% and 80% of full-term and preterm newborns, respectively [3].

In the Kingdom of Saudi Arabia, 15.9% of all newborns had indirect hyperbilirubinemia, of which ABO incompatibility was the most frequent cause, accounting for 31.6%, followed by G6PD (10.5%) and Rh incompatibility (2.6%) [4]. Most infants develop jaundice in the first week of their life, but the majority of cases are mild and unharmful [1]. Nevertheless, in approximately 8-10% of cases, NNJ can be severe and harmful to newborns [5]. Physiological jaundice is more likely to develop in breastfed infants in the first week of life. The main difficulty is differentiating rare cases of severe jaundice in babies, which can lead to bilirubin encephalopathy and kernicterus, from the majority of cases in which jaundice is mild and harmless [1]. NNJ has a significant impact on neonatal morbidity and mortality. Increased levels of unconjugated bilirubin in the blood can lead to significant complications such as kernicterus, cerebral palsy, acute bilirubin encephalopathy, seizures, deafness, and mental retardation [5]. Delays in seeking medical advice for NNJ can lead to severe hyperbilirubinemia, which contributes significantly to neonatal morbidity and mortality [5]. A study conducted among Saudi parents showed that 48.9% had a child with a history of NNJ, while only 43.8% had sufficient knowledge [5]. Another study performed in Arar City showed that 55% of the participants had a history of NNJ in their families and 49.9% had knowledge of NNJ [2].

On the other hand, a survey performed in Jazan showed that mothers have poor knowledge about NNJ [2]. In Ghana, the majority of mothers are aware of NNJ but have insufficient knowledge about its causes, danger signs, and treatment [6]. Surprisingly, healthcare workers in Nigeria have inadequate knowledge and misconceptions about NNJ [7]. Previous studies were conducted elsewhere on parents’ knowledge, attitudes, and practices regarding NNJ; to our knowledge, there has yet to be a study conducted among parents in Bisha. This study, therefore, aimed to determine the knowledge, attitudes, and practices of parents regarding NNJ in Bisha city.

## Materials and methods

This was a community-based cross-sectional study that aimed to determine the knowledge, attitudes, and practices of parents regarding NNJ. A sample size of 361 was calculated for the study, and a random sampling technique was used to recruit eligible participants from the population of Bisha City in the KSA. The study area comprised Bisha Province, which has a population of 204,491 and 240 villages [8].

The inclusion criteria for the study were parents who were capable of filling out an online questionnaire and living in Bisha, while parents living outside Bisha were excluded. The data were collected using an online questionnaire, which allowed respondents to select multiple items in certain questions. These questions have been previously validated by Alfouwais et al. [5].

Statistical analysis was carried out using RStudio (R version 4.2.2). Frequencies and percentages are used to present categorical data, whereas medians and interquartile ranges (IQRs) are used to express numerical variables. Statistical differences in the knowledge score between different groups were assessed using a Wilcoxon rank sum test for variables with two categories and a Kruskal-Wallis rank sum test for variables with three or more categories. The variables significantly associated with knowledge scores were subsequently used as independent variables in a multivariate generalized linear regression model to assess the independent predictors of knowledge. The results are presented as beta coefficients and their respective 95% confidence intervals (95% CIs). A p-value of <0.05 indicated statistical significance. Knowledge scores were computed by summing the scores for eight knowledge questions; each correct response was assigned a score of 1, and incorrect responses were assigned a score of 0. Therefore, the overall knowledge score ranged between 0 and 8.

Ethical clearance was obtained from the ethical committee at King Khalid University with number ECM#2023-805.

## Results

Demographic characteristics

Initially, we received 392 responses on the online platform. However, a total of 150 responses of those who were residing outside Bisha were excluded. Therefore, we analyzed the data of 242 participants. More than half of the participants were females (64.0%), employed (53.3%), and had received a postgraduate degree (62.0%). Approximately one-quarter of them were aged 20-30 years (25.2%) and 31-40 years (27.3%), whereas more than one-third of them had two to four children (38.0%, Table 1).

**Table 1:** Demographic characteristics

Parameter	Category	N (%)
Sex	Male	87 (36.0%)
	Female	155 (64.0%)
Age (years)	<20	27 (11.2%)
	20 to 30	61 (25.2%)
	31 to 40	66 (27.3%)
	41 to 50	58 (24.0%)
	>50	30 (12.4%)
Educational level	Illiterate	6 (2.5%)
	Primary	7 (2.9%)
	Preparatory	10 (4.1%)
	Secondary	69 (28.5%)
	Postgraduate	150 (62.0%)
Occupation type	Student	45 (18.6%)
	Employee	129 (53.3%)
	Unemployed	56 (23.1%)
	Retired	12 (5.0%)
Number of children	1 child	78 (32.2%)
	2 to 4 children	92 (38.0%)
	>4 children	72 (29.8%)

Responses to knowledge items

**Figure 1:**
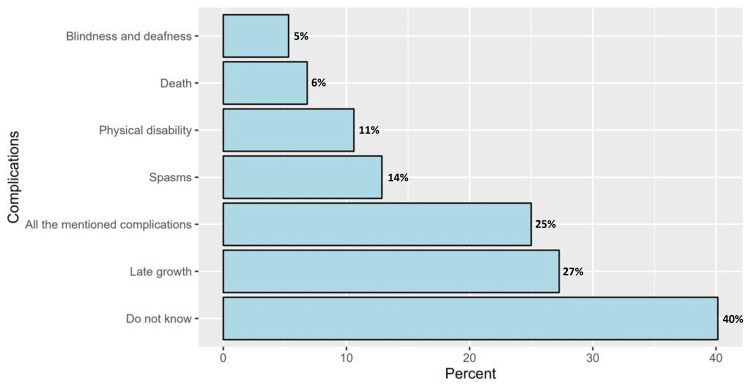
The proportions of complications of neonatal jaundice as perceived by the participants

Participants’ responses to knowledge items are demonstrated in Table 2. Regarding knowledge items, 45.0% and 26.4% of the respondents correctly defined NNJ as yellow pigmentation in the sclera of the eye and the skin, respectively. Additionally, the doctor was the ideal source of information regarding the disease among 14.5%. Child treatment in a hospital was the action of choice by 22.7% for a child suffering from NNJ, while 28.1% of them would do nothing. Out of the latter group, the majority of respondents did not know what to do, and 8.8% of them thought that jaundice is not a serious condition. Blue light was correctly identified by 33.9% as an effective light-curing modality, and the effect of sunlight was perceived as beneficial by 48.8% of them. Only 16.9% of respondents declared that regular examination after birth is the preventive method of choice for the condition. More than half of the participants (54.5%) indicated that NNJ may cause complications (Table 2). However, 40.2% of those who indicated the possibility of complications did not know about these unfavorable outcomes, whereas 27.3% and 25.0% indicated late growth and all the complications, respectively (Figure 1).

**Table 2:** Participants' responses to knowledge items *An asterisk indicates a correct answer

Parameter	Category	N (%)
Definition of neonatal jaundice	Yellow pigmentation in the sclera of the eyes (the whites of the eyes)*	109 (45.0%)
	Yellow pigmentation of the skin*	64 (26.4%)
	Change in the color of urine or stool	29 (12.0%)
	I do not know	99 (40.9%)
Source of knowledge about neonatal jaundice	No source	101 (41.7%)
	The doctor who treated my child*	35 (14.5%)
	Friends and relatives	69 (28.5%)
	Social media	37 (15.3%)
How was your behavior when your child had neonatal jaundice?	I did not do anything	68 (28.1%)
	Increased breastfeeding	38 (15.7%)
	Began providing external milk	8 (3.3%)
	Sought treatment for the child in a hospital*	55 (22.7%)
	Gave the child antibiotics	7 (2.9%)
	Gave the child herbal medications	6 (2.5%)
	Exposed the child to sunlight	35 (14.5%)
	Exposed the child to neon light (light in the house)	25 (10.3%)
Why did you not seek treatment for your child in a hospital?	I do not know	58 (85.3%)
	Neonatal jaundice does not require treatment	1 (1.5%)
	I think neonatal jaundice is not serious	6 (8.8%)
	Because blood samples need to be taken for neonatal jaundice	0 (0.0%)
	Fear of hospitals	1 (1.5%)
	Herbal medications are better	2 (2.9%)
Which type of light-curing treatment is effective?	White	115 (47.5%)
	Blue*	82 (33.9%)
	Green	45 (18.6%)
What is the effect of sunlight on neonatal jaundice?	I do not know	71 (29.3%)
	Neither beneficial nor harmful	30 (12.4%)
	Harmful	23 (9.5%)
	Beneficial*	118 (48.8%)
Neonatal jaundice may cause complications	No	110 (45.5%)
	Yes*	132 (54.5%)
How can neonatal jaundice be prevented?	I do not know	90 (37.2%)
	Prenatal examination and follow-up	64 (26.4%)
	Regular examinations after birth*	41 (16.9%)
	Previous knowledge about neonatal jaundice	23 (9.5%)
	Following a healthy diet during pregnancy	16 (6.6%)
	Preventing infection	8 (3.3%)

Characteristics of neonatal jaundice and participants' attitudes toward the condition

In general, 59 parents (24.4%) had a history of a child with NNJ, of whom 54.4% had one child with the disease, 31.6% had two children with the disease, and 14.0% had more than two children with the disease. Of note, 36.8% of the respondents indicated that they had sufficient knowledge about NNJ. A great proportion of them (68.2%) claimed that they had knowledge despite having no children with the disease, while only 21.5% declared that they acquired the knowledge after their child got the disease. As reported by the participants, the best way to educate people about the condition is to create awareness campaigns (40.5%) and through social networking programs (33.5%, Table 3).

**Table 3:** Characteristics of neonatal jaundice and participants' attitudes toward the condition

Parameter	Category	N (%)
Do you have a child with a history of neonatal jaundice?	No	183 (75.6%)
	Yes	59 (24.4%)
If yes, how many children?*	1 child	31 (54.4%)
	2 children	18 (31.6%)
	>2 children	8 (14.0%)
Time at which knowledge about neonatal jaundice was attained	I have knowledge and my child was not diagnosed with neonatal jaundice	165 (68.2%)
	Before my child was diagnosed with neonatal jaundice	25 (10.3%)
	After my child was diagnosed with neonatal jaundice	52 (21.5%)
The best way to educate and stimulate awareness about neonatal jaundice	Create public awareness campaigns about neonatal jaundice	98 (40.5%)
	Through social networking programs	81 (33.5%)
	Provide brochures to mothers during prenatal care visits	63 (26.0%)

Factors associated with the scores of knowledge

For the whole sample, the median (IQR) knowledge score was 3.0 (1.0-4.0) with a minimum of 0 and a maximum of 7. Results of the statistical differences showed that females had significantly higher knowledge scores than males (median = 3.0; IQR = 2.0-4.0 vs median = 2.0; IQR = 1.0-3.5; p = 0.021), among those who reported sufficient knowledge about the disease (median = 4.0; IQR = 3.0-5.0 vs median = 2.0; IQR = 1.0-3.0 among those who had no sufficient knowledge; p < 0.001), and those who had a child with NNJ (median = 4.0; IQR = 3.0-4.0 vs median = 2.0; IQR = 1.0-3.0 among those who had no children with NNJ; p < 0.001). Additionally, participants who had acquired their knowledge after a child had had NNJ had a significantly higher knowledge score (median = 4.0; IQR = 3.0-4.3) compared to those who had acquired their knowledge before a child had had NNJ (median = 3.0; IQR = 2.0-4.0) and those who had no children with the disease (median = 2.0; IQR = 1.0-4.0; p < 0.001; Table 4). On the multivariate analysis, higher knowledge scores were independently associated with the female gender (beta = 0.54; 95% CI, 0.16 to 0.91; p = 0.005) and having sufficient knowledge about NNJ (beta = 1.59; 95% CI, 1.19 to 1.98; p < 0.001; Table 5).

**Table 4:** Statistical differences in the knowledge scores regarding neonatal jaundice

Parameter	Category	Median (IQR)	p-value
Sex	Male	2.00 (1.00, 3.50)	0.021
	Female	3.00 (2.00, 4.00)	
Age (years)	<20	2.00 (1.00, 3.00)	0.462
	20 to 30	3.00 (1.00, 4.00)	
	31 to 40	2.50 (1.00, 4.00)	
	41 to 50	3.00 (1.00, 4.00)	
	>50	3.00 (2.00, 4.00)	
Educational level	Illiterate	2.00 (1.25, 2.00)	0.853
	Primary	2.00 (2.00, 4.00)	
	Preparatory	2.00 (2.00, 3.00)	
	Secondary	3.00 (1.00, 4.00)	
	Postgraduate	3.00 (1.00, 4.00)	
Occupation type	Student	3.00 (1.00, 4.00)	0.144
	Employee	2.00 (1.00, 4.00)	
	Unemployed	3.00 (2.00, 4.00)	
	Retired	3.50 (2.00, 4.25)	
Number of children	1 child	2.50 (1.00, 4.00)	0.611
	2 to 4 children	3.00 (1.75, 4.00)	
	>4 children	3.00 (1.00, 4.00)	
Have sufficient knowledge about neonatal jaundice	No	2.00 (1.00, 3.00)	<0.001
	Yes	4.00 (3.00, 5.00)	
Have a child with a history of neonatal jaundice	No	2.00 (1.00, 3.00)	<0.001
	Yes	4.00 (3.00, 4.00)	
Time at which knowledge about neonatal jaundice was attained	I have knowledge and my child was not diagnosed with neonatal jaundice	2.00 (1.00, 4.00)	<0.001
	Before my child was diagnosed with neonatal jaundice	3.00 (2.00, 4.00)	
	After my child was diagnosed with neonatal jaundice	4.00 (3.00, 4.25)	

**Table 5:** Independent predictors of knowledge among the participants

Parameter	Category	Beta	95% CI	p-value
Sex	Male	-	-	
	Female	0.54	0.16, 0.91	0.005
Do you have sufficient knowledge about neonatal jaundice?	No	-	-	
	Yes	1.59	1.19, 1.98	<0.001
Do you have a child with a history of neonatal jaundice?	No	-	-	
	Yes	0.24	-0.38, 0.86	0.449
When did you receive knowledge about neonatal jaundice?	I have knowledge and my child was not diagnosed with neonatal jaundice	-	-	
	Before my child was diagnosed with neonatal jaundice	-0.4	1.00, 0.25	0.227
	After my child was diagnosed with neonatal jaundice	0.55	-0.09, 1.20	0.094

## Discussion

Our study aimed to assess the knowledge and attitudes of parents regarding NNJ in the Bisha region. The findings revealed several key results related to demographic characteristics, knowledge levels, attitudes toward the condition, and factors associated with knowledge scores.

In terms of demographic characteristics, the study analyzed data from 242 participants who resided in Bisha. The majority of participants were female, employed, and had a postgraduate degree. This demographic profile suggests that the study sample comprised educated individuals, which may have influenced their knowledge and attitudes toward NNJ.

Our study demonstrated a varied level of knowledge among respondents regarding NNJ and its management. In terms of defining NNJ, a moderate proportion of participants correctly identified yellow pigmentation in the sclera of the eye (45.0%) and the skin (26.4%) as characteristic features of the condition. These findings align with a similar study conducted by Demis et al. (2021), where approximately 39.2% of respondents accurately defined NNJ [9].

Interestingly, only 14.5% of participants considered a doctor as the ideal source of information for NNJ. This finding contrasts with the study by Salia et al. (2021), which reported a higher preference for healthcare professionals as the preferred source of information among parents [10]. The disparity in these results may be attributed to differences in sample characteristics or cultural factors influencing health-seeking behaviors and information sources.

Regarding the preferred action for managing NNJ, the study revealed that 22.7% of parents preferred child treatment in a hospital, while 28.1% opted for doing nothing. It is worth noting that a significant portion of parents who chose to do nothing cited a lack of knowledge or awareness of appropriate actions, while 8.8% perceived jaundice as a non-serious condition. These findings are consistent with a study by Ogunfowora and Daniel (2006), which found that a considerable number of parents lacked knowledge about the management of NNJ and its potential complications [7].

In terms of treatment modalities, 33.9% of respondents correctly identified blue light as an effective light-curing method, while 48.8% perceived sunlight as beneficial for the condition. These findings align with the study conducted by Wang et al. (2021), which reported a similar proportion of parents recognizing blue light as a treatment option for NNJ [11]. The perception of sunlight as a beneficial modality also aligns with previous studies for the treatment and prevention of NNJ [12].

Furthermore, only 16.9% of participants indicated that a regular examination after birth was the preferred preventive method for NNJ. This finding is consistent with the study by Amegan-Aho et al. (2019), which highlighted a lack of awareness among parents regarding the importance of regular postnatal check-ups for early detection and management of NNJ [6].

Regarding the awareness of complications associated with NNJ, more than half of the participants (54.5%) recognized the potential for complications. However, among those who indicated the possibility of complications, 40.2% were unaware of the specific unfavorable outcomes. This finding aligns with a study by Magai et al. (2020), which revealed a lack of knowledge about the potential long-term consequences of NNJ among parents [13].

The findings of this study provide valuable insights into the characteristics of NNJ and the attitudes of parents toward this condition. Approximately 24.4% of the participants reported having a child with a history of NNJ, indicating that it is a relatively common condition among newborns. Interestingly, the majority of parents (54.4%) reported having only one child with NNJ, while 31.6% had two children, and 14.0% had more than two children affected by the condition. These results highlight the recurrence of NNJ within families, suggesting a possible genetic predisposition or shared environmental factors [14].

In terms of knowledge about NNJ, the study found that 36.8% of respondents believed they had sufficient knowledge about the condition. It is noteworthy that a significant proportion of parents (68.2%) claimed to have knowledge about NNJ despite not having any children affected by the condition. This indicates that parents may acquire information about NNJ through sources other than personal experience, such as healthcare professionals, educational materials, or community awareness campaigns [7,15].

Interestingly, only 21.5% of parents reported acquiring knowledge about NNJ after their child was diagnosed with the condition. This finding suggests that there may be a lack of proactive education and awareness regarding NNJ, as parents tend to seek information after their child’s diagnosis rather than proactively seeking knowledge about the condition beforehand. This highlights the importance of early education and awareness campaigns to ensure parents are well-informed about NNJ before it affects their children [15].

The study also investigated the participants’ attitudes toward educating others about NNJ. According to the respondents, the most effective ways to educate people about the condition were through awareness campaigns (40.5%) and social networking programs (33.5%). These findings emphasize the role of public health initiatives in raising awareness and disseminating information about NNJ [16,17]. Awareness campaigns can reach a large audience and provide comprehensive information about the condition, while social networking programs can leverage the power of social media platforms to share knowledge and experiences among parents and caregivers.

Regarding the factors associated with knowledge scores regarding NNJ. The median knowledge score for the entire sample was 3.0, with a range of 0-7. Several key findings emerged from the analysis, highlighting factors that were significantly associated with higher knowledge scores.

One important finding was that females exhibited significantly higher knowledge scores compared to males. This finding aligns with previous studies that have reported a gender difference in health knowledge [18]. It is plausible that females, in general, might have greater exposure to healthcare information or have higher health-seeking behaviors, leading to better knowledge about NNJ.

Another significant factor associated with higher knowledge scores was having sufficient knowledge about the disease. Participants who reported having sufficient knowledge had significantly higher knowledge scores compared to those with insufficient knowledge. This finding emphasizes the importance of adequate health education and information dissemination about NNJ. It suggests that when individuals have access to accurate and comprehensive information, their knowledge levels are likely to be higher. This finding is consistent with previous studies that have shown the positive impact of health education programs on knowledge improvement [19].

Furthermore, participants who had a child with NNJ demonstrated significantly higher knowledge scores compared to those who did not have a child with the condition. This result implies that personal experiences, such as witnessing and managing NNJ in their own child, may contribute to a deeper understanding of the disease. Similar findings have been reported in previous studies examining the impact of personal experiences on knowledge acquisition [20].

Interestingly, participants who acquired knowledge after their child was diagnosed with NNJ exhibited significantly higher knowledge scores compared to those who acquired knowledge before having a child with the condition or those who had no children with NNJ. This finding suggests that firsthand experience and exposure to the disease might have a more profound effect on knowledge acquisition and retention. It is possible that individuals actively seek information when faced with a health issue affecting their child, leading to a more comprehensive understanding of the condition.

In the multivariate analysis, the female sex and having sufficient knowledge about NNJ were independently associated with higher knowledge scores. These results further confirm the significant impact of gender and health education on knowledge levels. Females, even after controlling for other factors, maintained higher knowledge scores compared to males, suggesting a consistent gender disparity in health knowledge. Moreover, participants who reported having sufficient knowledge remained significantly associated with higher knowledge scores, highlighting the importance of targeted health education interventions to improve knowledge about NNJ.

It is important to acknowledge the limitations of this study. The findings are based on a specific sample from the Bisha region, which may not be representative of the entire population. The reliance on self-reported data introduces the possibility of recall bias or social desirability bias. Future studies should aim for larger and more diverse samples to provide a more comprehensive understanding of parental knowledge and attitudes toward NNJ in Saudi Arabia.

## Conclusions

In conclusion, this research has shed light on various aspects of parental knowledge and attitudes toward NNJ in the Bisha region. A notable proportion of parents expressed uncertainty about appropriate actions, reflecting the knowledge gap that necessitates attention. The study findings underscore the importance of tailored health education initiatives aimed at both genders, emphasizing the potential consequences and management of NNJ.
